# Statistical Treatment of Looking-Time Data

**DOI:** 10.1037/dev0000083

**Published:** 2016-02-04

**Authors:** Gergely Csibra, Mikołaj Hernik, Olivier Mascaro, Denis Tatone, Máté Lengyel

**Affiliations:** 1 Department of Cognitive Science, Central European University, and Department of Psychological Sciences, Birkbeck, University of London; 2 Department of Cognitive Science, Central European University; 3 Computational and Biological Learning Lab, Department of Engineering, University of Cambridge, and Department of Cognitive Science, Central European University

**Keywords:** looking times, Bayesian statistics, infancy, log-normal distribution

## Abstract

Looking times (LTs) are frequently measured in empirical research on infant cognition. We analyzed the statistical distribution of LTs across participants to develop recommendations for their treatment in infancy research. Our analyses focused on a common within-subject experimental design, in which longer looking to novel or unexpected stimuli is predicted. We analyzed data from 2 sources: an in-house set of LTs that included data from individual participants (47 experiments, 1,584 observations), and a representative set of published articles reporting group-level LT statistics (149 experiments from 33 articles). We established that LTs are log-normally distributed across participants, and therefore, should always be log-transformed before parametric statistical analyses. We estimated the typical size of significant effects in LT studies, which allowed us to make recommendations about setting sample sizes. We show how our estimate of the distribution of effect sizes of LT studies can be used to design experiments to be analyzed by Bayesian statistics, where the experimenter is required to determine in advance the predicted effect size rather than the sample size. We demonstrate the robustness of this method in both sets of LT experiments.

Measuring looking times (LTs) is one of the most frequently used behavioral techniques in research with infants. LTs are measured and evaluated in various paradigms, which share the basic assumption that the length of time for which infants look at a stimulus reflects the operation of the psychological mechanisms recruited to process it. For example, many studies were devoted to the analysis of habituation: how LTs to the same stimulus change across successive trials and whether the individual differences in this habituation function reflect stable characteristics of infants and/or predict their later achievements (e.g., Colombo, 2001; Dannemiller, 1984; Gilmore & Thomas, 2002). Another use of LTs is the measurement of variability of duration of looks during free viewing of continuous dynamic stimuli, such as TV programs (e.g., Richards, 2010; Richards & Anderson, 2004). LTs, or their proportion, can be compared between stimuli presented simultaneously (e.g., Ferry, Hespos & Waxman, 2010; Senju & Csibra, 2008).

In the context of this article we restrict our analysis to one particular utilization of LTs: the study design in which infants are successively exposed to two different stimuli, the LT to these stimuli are measured, and the difference of looking duration is interpreted in terms of the underlying cognitive mechanisms. Within-subject comparison of LTs is among the most popular study designs in infancy research, not only because it requires fewer participants than the between-subjects design, but also because it efficiently deals with the fact that baseline LTs vary considerably across infants (Gilmore & Thomas, 2002). Paradigms adopting this design include the assessment of visual preference between two stimuli, evaluating the effect of familiarity or novelty after familiarizing infants with, or habituating them to, certain stimuli, and testing whether infants develop expectations about states of affairs in a situation by presenting them with events that would confirm or violate these expectations (e.g., Baillargeon, Spelke, & Wasserman, 1985; Kellman & Spelke, 1983; Woodward, 1998; Wynn, 1992; Younger & Cohen, 1983). LTs to visual stimuli are also measured to assess infants’ processing of accompanying auditory stimuli or multimodal matching (e.g., Gogate & Bahrick, 1998; Kuhl & Meltzoff, 1984; Saffran, Aslin, & Newport, 1996; Werker, Cohen, Lloyd, et al., 1998; Yeung & Werker, 2009).

Depending on what mechanisms and what experience researchers consider relevant in a situation, there could be different ways of interpreting the results of a study measuring LTs in this kind of paradigm (Bogartz, Shinskey, & Speaker, 1997; Houston-Price & Nakai, 2004; Rivera, Wakeley, & Langer, 1999; Roder, Bushneil, & Sasseville, 2000; Slater, 2004). In this article, we are not concerned with these controversies (Aslin, 2000; Cohen, 2004; Haith, 1998; Munakata, 2000; Sirois & Mareschal, 2002). Instead, our aim is to evaluate the quantitative nature of LT measures and offer recommendations for their efficient use in infant research. First, we establish that LTs are log-normally distributed across participants, and therefore, need to be log-transformed before parametric statistical analysis. Second, we estimate the typical size of significant effects in LTs studies, which allows us to make recommendations about setting sample sizes. Third, our estimate of the distribution of effect sizes of LT studies can be used in Bayesian statistics, which have several important advantages over standard “frequentist” statistical tests (Dienes, 2011, 2014; Howard, Maxwell, & Fleming, 2000; Kruschke, 2013; Sanborn & Hills, 2014; van de Schoot, Kaplan, Denissen, et al., 2014; Wagenmakers, 2007).

It is often reported that LTs show a right-skewed distribution across participants (e.g., Farroni et al., 2005; Garnham & Ruffman, 2001; Leslie & Chen, 2007), because, within a specific experiment, the infants who look longer than average to the stimulus produce LTs that tend to be farther away from the mean than the LTs of short-lookers. Strong skewness of LT data renders them formally inappropriate for parametric statistical analyses, such as analysis of variance (ANOVA), *t* test and regression analysis, which require normally distributed data, especially with small sample sizes (8 to 32 participants) characteristic of LT studies. One way to deal with this problem is to treat extreme long-lookers as outliers and to exclude them from the sample on the basis of some statistical criteria (e.g., Beier & Spelke, 2012; Sommerville, Hildebrand, & Crane, 2008; Surian, Caldi, & Sperber, 2007; Wagner & Carey, 2005). While such exclusions may be justified in many cases, sometimes the criteria applied to them seem to be ad hoc and arbitrary, and, in general, discarding data is detrimental to statistical efficiency. Another way to deal with a skewed distribution is to apply some type of, most frequently logarithmic, transformation to the data before statistical analysis (e.g., Csibra, 2001; Farroni, Csibra, Simion, et al., 2002; Kibbe & Leslie, 2011; Kirkham, Slemmer, & Johnson, 2002; Starkey, Spelke, & Gelman, 1990; Woodward, 1998). This method is usually justified by statistical tests that demonstrate the nonnormality of the distribution of raw LTs.

These two kinds of procedures treat the skewed distribution of LTs across infants as a statistical accident that has to be corrected before proper analysis of the data could begin. However, another possibility is that the distribution of LTs across participants *systematically* deviates from normality, whether or not this is evident, or statistically demonstrable, in experiments conducted with the small sample size that is typical in studies with infants. LTs are typically assessed from a specific moment in time that determines the onset of the psychological process under investigation (e.g., the onset of the stimulus to be detected, accomplishment of an event-outcome to be interpreted, or the moment when stimuli start to diverge across conditions), and the same is true of many other dependent measures of duration, such as reaction times (RTs). Duration in these types of measurements is a nonnegative quantity with a nonarbitrary zero point, and it can as much be considered to be on a ratio scale as on an interval scale. Interpreting LTs proportionally to each other makes intuitive sense: an infant who is fast in processing a stimulus may display 10% (rather than, say, 1 s) shorter looking times than others, and a difficult event may protract looking by 40% rather than by a fixed amount of time (say, 4 s). Thus, it is possible, or even plausible, that the factors influencing the duration of looking measured from a specific zero point are not additive but multiplicative in nature. If this is the case, then LTs would be better considered to be the product than the sum of different independent factors, and, according to the central limit theorem, their distribution would follow a log-normal rather than a normal distribution.

Indeed, the duration of individual looks toward a TV screen was reported to be distributed log-normally within infants (for a review, see Richards, 2010). The phenomenon that the longer a look has been lasting for the less likely it is terminated was attributed to the deepening of attentional engagement during a look, resulting in “attentional inertia” and a characteristic distribution of individual looks. However, the log-normal distribution is not restricted to LTs but is also characteristic of RTs (Ulrich & Miller, 1993; Woodworth & Schlosberg, 1954) and other duration-based psychological phenomena (e.g., Zhou et al., 2004), and is manifest in distributions not only within subjects but also across subjects. In fact, such log-normal distributions are ubiquitous across several disciplines (for a review, see Limpert, Stahel, & Abbt, 2001).

A random variable is log-normally distributed if its logarithm is normally distributed. This distribution assumes only positive values, and is always right-skewed, though the amount of skewness depends on the actual parameters. If LTs are log-normally distributed then it is the rule rather than the exception that their distribution is skewed, and logarithmically transformed LTs should generally approximate normal distribution better than raw data. However, a single looking-time study cannot provide sufficient amount of data to confirm or reject the hypothesis that LTs are log-normally distributed. So far, systematic analyses of statistical properties of LTs investigated how LTs measured from the same individuals (in free viewing or across habituation trials) are distributed across measurements (e.g., Pempek et al., 2010; Richards & Anderson, 2004; Thomas & Gilmore, 2004). However, to test our hypothesis, we needed a different kind of information, namely how LTs are distributed among different individuals and across studies. To obtain this information, we analyzed two different sets of data: a set of studies conducted by members of our laboratory during recent years, and a representative set of published studies from the infant research literature.

## Data Sets

We performed our analyses on two sets of data: an in-house data set, and a set of published studies (all data are available as an Excel file in the Supplementary Material). LTs were measured in, or transformed to, seconds in all cases. Note that although we could include in the in-house data set unsuccessful, and eventually unpublished, experiments, the data set collected from the literature was more likely biased by the so-called “file-drawer problem” (Rosenthal, 1979).

### In-House Data Set

We combined the data from 13 studies, comprising 47 experiments, which included all the completed LT experiments conducted by the second, third, and fourth author of this article, irrespective of their results (see Table 1). In each of these experiments, infants between 6 and 15 months of age were familiarized with (45 experiments) or habituated to (2 experiments) video-taped or animated events presented on a computer screen, and their LTs were measured in two subsequent test events (representing two conditions) to single (not paired) stimuli, counterbalanced in order. The data set included the LTs to these 94 test events from all participants. The prefixed sample size in each experiment was either 16 or 24 (after exclusion of participants for fussiness, failure to look for a predetermined minimum amount of time or at crucial events during familiarization or test, parental interference, technical failure, or experimental error). In some of the experiments we had predicted a difference between the LTs to the two test events (which was not always confirmed), in others we did not (control experiments). Some of these studies have already been published, others are under review, still others are in progress with further conditions being added to them, or will remain unpublished. We do not report either the hypotheses or the specifics of these experiments, because we are only concerned here with the statistical properties of the collected data.[Table-anchor tbl1]

The average of the mean LTs calculated across participants within the 94 conditions (two conditions in each experiment) in the in-house data set was 14.6 s, with the average *SD*s of 9.6 s. The age group of the infants (averaged to 12.6 months) did not correlate with the mean or *SD* across these conditions (*r* = .090 and 0.081, respectively; *p* > .3).

### Literature Data Set

We identified articles to be included in our study through a search by Google Scholar for relevant articles. The search, performed in October 2013, used the exact query: *infant OR infants “looking time” OR “looking times”* and restricted the results to articles tagged as published in 2012. This query yielded 639 results. Within this set we collected data from all available studies that (a) were published in a peer-reviewed journal (note that some of the articles had the eventual publication date of 2013), and (b) measured LTs in infants not older than 24 months. Moreover, to be included, articles (c) had to report infants’ LTs at a scene until a criterion was reached (typically up to the point participants looked away for a preset amount of time, or reached a maximum amount of looking). Other measures of LTs (e.g., comparing amounts of looking at two different areas of a single scene) were not included. (d) We included only articles reporting within-subject LT data for two comparable types of test events (e.g., consistent and inconsistent tests). We chose to focus on within-subject designs because they are the most commonly used in infants’ LTs studies (in our sample, only three articles used between-subjects designs to assess the effect of condition on infants’ LTs). (e) We also excluded articles that did not provide sufficient information to extract relevant data (participants’ age, and means and *SD*s of LTs).

The final data set included 33 articles (149 experiments altogether) reporting data from infants 2 to 24 months of age, and covering a large span of presentation modes, methods, and topics (see Table 2). Relevant data was collected primarily from the text of the articles. However, when means and *SD*s of LTs were not available in the text, we extracted them from graphs, estimating means and standard errors in seconds to 1 decimal digit precision. The literature data set was collected by three independent coders, who each processed about one third of the articles.[Table-anchor tbl2]

The average of the means and the SDs of the 298 conditions were 11.2 s and 6.4 s, respectively, and neither statistic was correlated with the age group (*r* = .082 and 0.020, respectively; *p* > .15). However, infants in the experiments of the literature data set were significantly younger (mean 9.6 months) than those of the in-house data set (*t*_194_ = 3.656, *p* < .001), and produced shorter mean LTs (*t*_390_ = 3.935, *p* < .001) and corresponding SDs (*t*_390_ = 6.024, *p* < .001). We also investigated whether these statistics differed between purely visual experiments (*n* = 88) and those that measured LTs to auditory stimuli or to audio-visual matching (*n* = 61) in the literature data set. Visual experiments produced longer mean LTs (13.0 s) and *SD* of LTs (7.7 s) than auditory studies (8.5 and 4.5 s, respectively), and these differences were statistically significantly across the 298 conditions (*t*_296_ = 4.943 and 5.582, respectively, *p* < .001 in both cases).

## The Distribution of Looking Times

The distribution of LTs can be analyzed both within a study (across individuals) and across studies. Because the first method requires access to individual data, we applied it only to our in-house data set.

### Within-Study Distribution

**Figure 1 fig1:**
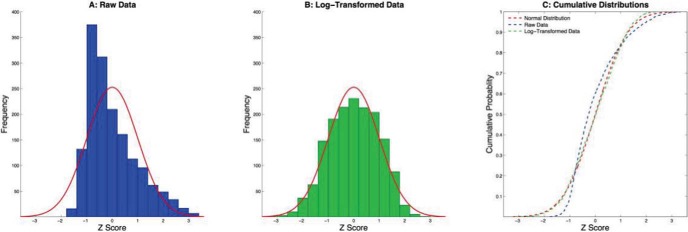
Distribution of standardized looking times (LTs) (converted to Z scores within conditions) in the in-house data set compared with the standard normal distribution. (A) Histogram derived from raw data. (B) Histogram derived from log-transformed data. (C) Cumulative distribution of raw, and log-transformed data and the normal distribution. On A and B, the area below the Gaussian curve is equal to the total area of the histogram. See the online article for the color version of this figure.

In our in-house data set, we measured LTs to two test events in each of the 47 experiments. Thus, we had 94 sets of LT data (representing 94 experimental conditions) comprising 1,584 observations (16 or 24 measurements per condition). Assuming that the data within each condition was normally distributed, these sets would be different only in scale and their mean. We standardized the data within each set by subtracting its own sample mean and dividing the results with the sample *SD*. If the sampled data within each condition were coming from a normal distribution, the standardized data in each study should come from the same standard normal distribution with mean 0 and *SD* 1. We then collapsed the data across all conditions to check how well the sample of the 1,584 standardized observations fitted the standard normal distribution. Figure 1A shows a histogram of our finding contrasted with a standard normal distribution density function.[Fig-anchor fig1]

It is clear from this figure that the match between the standardized data and the standard normal distribution is rather poor. Although the mean of the collapsed standardized data is 0 (by definition), the mode is closer to −1, and the distribution is heavily skewed (skewness = 0.97). According to an often used 2.5 *SD* criterion for identifying outliers, 29 data points (1.83%) would be excluded from these data as outliers in their respective conditions, which would result in only a slight change to the skewness (0.82).

Next, we checked the hypothesis that our data were coming from log-normal distributions. We log-transformed all 1,584 data points, and then standardized the data within each condition the same way as we did with the raw data. The collapsed data set is depicted on Figure 1B as a histogram against the standard normal distribution. The distribution of standardized log-transformed data is only slightly skewed to the left (skewness = −0.11) and its shape matches closely that of the normal distribution. Only 6 data points (5 on the left and 1 on the right) would be excluded from the log-transformed data with a 2.5 *SD* criterion of outliers.

The distributions of the raw and the log-transformed data can be directly compared by their cumulative distributions (Figure 1C). According to Kolmogorov–Smirnov tests, the distribution of raw standardized LTs was significantly different from standard normal distribution (*p* < .0001) while the distribution of the log-transformed LTs was not (*p* = .1354). We conclude that the distribution of individual raw LTs within each condition was likely log-normal rather than normal.^1^

### Across-Study Distribution

While the above analysis investigated the distribution of LTs across infants within individual conditions, it does not reveal the factors that affect this measure across studies. However, if these factors, just like individual differences, exert multiplicative effects on LTs, one may hypothesize that the distribution of LTs across studies will differ mainly in scale. (Note that this hypothesis is theoretically independent from the within-study distribution of LTs.) One consequence of this hypothesis is that within-Condition SDs should change proportionally to the means, while the coefficients of variation should remain relatively constant.

#### In-house data set

**Figure 2 fig2:**
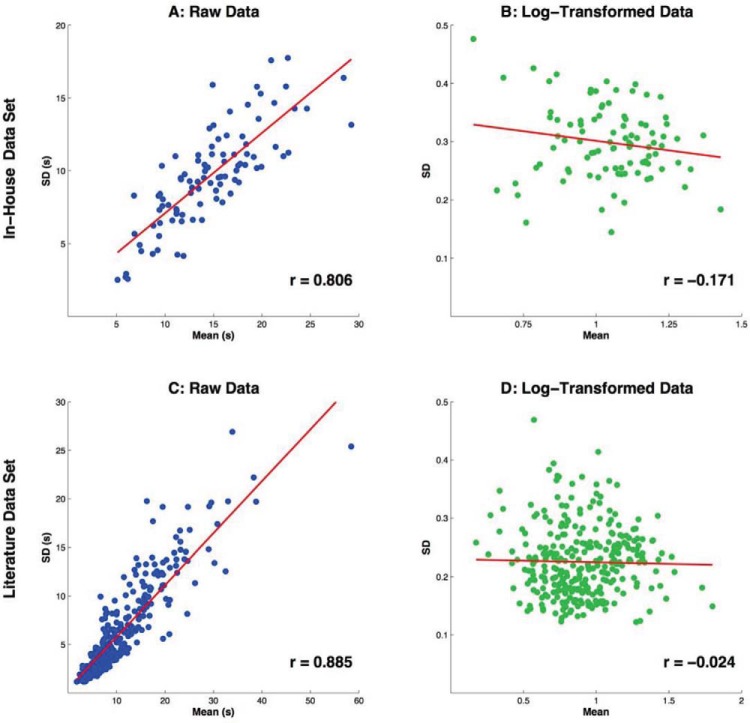
Scatterplots illustrating the relationship between means and *SD*s of looking time (LT) data of the two data sets with raw and log-transformed forms. The lines represent the best-fit linear regression. See the online article for the color version of this figure.

Figure 2A depicts the relationship between the means and *SD*s in the 94 sets of LTs in the in-house data set: the correlation between means and *SD*s is high (above 0.8), as the *SD* tends to increase linearly with the mean across studies and conditions. Specifically, the *SD* for each set of data tended to be about 2/3 of the mean (the average coefficient of variation across conditions was 0.667).[Fig-anchor fig2]

Logarithmic transformation removed this scaling factor: the means and *SD*s of (base-10) logarithmically transformed data were barely correlated (Figure 2B). In addition, the coefficient of variation of *SD*s across conditions was 0.35 in the raw and 0.21 in the transformed data, suggesting more homogeneity after transformation. The majority of *SD*s in the log-transformed data was between 0.25 and 0.35, with the average *SD* being 0.298. We conclude that, in the in-house data set, LTs displayed log-normal distributions, with the underlying normal distributions differing in mean (corresponding to differences in average log-looking time), but had a relatively uniform *SD*, which was close to 0.3.

#### Literature data set

The correlation between means and *SD*s of LTs was even stronger in the sample of 298 conditions collected from the literature (Figure 2C), with the average coefficient of variation being lower than in the in-house sample, 0.566.

Assuming that the LTs in all studies in this data set were coming from log-normal distributions, we estimated the means and SDs of the base-10 log-transformed data from the means and *SD*s of the raw data (for the details of the calculation, see Appendix A). In the transformed data set, the correlation between means and *SD*s completely disappeared (Figure 2D). This outcome is consistent with the assumptions that LT distributions within studies are log-normal and the factors influencing LT differences across studies tend to be multiplicative.

### Interpretation

Log-transformation of LT data is thus recommended before parametric statistical analyses are performed because these analyses usually assume (a) normal distribution, and (b) homogeneity of variance (though with appropriate choice of tests, *t* tests and ANOVAs are robust with respect to the latter condition). If LTs are log-normally distributed and mainly differ in scale across conditions, then both of these assumptions are violated by raw data but met by log-transformed data. It is important to note that parametric multifactor analyses of LTs could also result in spurious effects (e.g., showing an interaction rather than two main effects in an ANOVA) if they are applied to raw data.

## Statistical Comparison of LTs Across Conditions on Raw Versus Log-Transformed Data In-House Data Set

When we analyzed the raw LTs in the in-house data set by within-subject *t* tests at α = 0.05, 15 of the 47 experiments showed statistically significant differences between conditions (14 in the expected direction and one in the opposite direction). Using log-transformed LTs, 19 experiments produced statistically significant effects (18 in the expected direction, 1 in the opposite direction), 14 of them being the same experiments as with nontransformed data. Thus, log-transformation of LTs revealed statistically significant effects in five experiments that would have been rendered nonsignificant by analyses of nontransformed data. Only one experiment rendered significant effect with raw LTs but not with log-transformed data. While log-transformation does not necessarily increase the chance of obtaining significant results, when the data are skewed and the LT difference between test trials is small, it could reveal an otherwise hidden effect.

### Literature Data Set

To perform statistical tests on these data, we needed the *SD* of the within-subject, between-condition differences of log-transformed LTs for each experiment. While this information was not directly available to us, we could still estimate it using available information about the statistics of raw LTs. First, we recovered the between-condition Pearson correlation of the raw LTs from the corresponding *t*-values, whenever these were reported (where, instead of *t* tests, one-way ANOVAs were performed, we used the reported *F* values to calculate the corresponding *t*-values; see Appendix B for the details of the calculation). In the 64 (of 149) experiments that reported *t*-statistics (or allowed us to calculate *t*-values), the average between-condition correlation of raw LTs was *r* = .421, very close to what we found in the in-house data set (*r* = .414). This allowed us, in a second step, to estimate the between-condition correlation of the log-transformed LTs in these 64 experiments by using the regression equation we found in our in-house data set relating correlations of log-transformed and raw LT values.^2^ Third, we assumed that the average correlation coefficient between log-transformed LTs in these 64 experiments (*r* = .386) was representative, and applied this value to the remaining 85 experiments. Finally, we estimated the *SD* of the log-transformed LT differences using the correlation values obtained above in the whole data set (see Equation B2 in Appendix B).

Eighty-four of the 149 experiments in this data set were reported to produce a statistically significant effect based on the analysis of the raw data (including one where the effect went in the unpredicted direction), and 14 of these experiments were judged to be nonsignificant with the estimated log-transformed data. Seven of these 14 mismatches might have been because of the fact that we had to resort to estimating correlations between LTs when *t* tests or one-way ANOVAs were not reported for them (see above). In all these seven cases, the raw LTs must have been very strongly correlated (0.72, 0.74, 0.79, 0.86, 0.87, 0.92, and 0.98), otherwise they could not have accounted for the effect. The other seven experiments produced *p* values only slightly below the 0.05 level, and log-transformation pushed them above that level. However, in further two experiments log-transformation made the statistical results stronger, turning one-tailed significant effects into two-tailed ones.

### Statistical Parameters of Log-Transformed Looking Times

In the following, unless we specify otherwise, our analyses are based on log-transformed LT data (or their estimates), which we assume to have an approximately normal distribution. For parametric statistical analyses of within-subject data with a normal distribution, only three summary statistics of the sample need to be taken into account: the sample size, and the mean and *SD* of the between-condition, within-subject differences. The sample sizes were given in the data sets. The *SD* of the within-subject differences depends on the *SD* of the LTs and the correlation between the LTs in the two conditions. (At *r* = .5, the *SD* of the difference would be about the same as the SDs of the LTs as long as they do not differ substantially from each other.) The difference between the LTs of the two conditions represents the effect produced by the experimental manipulation. Where this manipulation had the predicted effect, we expect to see a positive value; where it did not have an effect, the value will be close to zero. We investigated the distribution of these two parameters (*SD* and mean of between-Condition LT differences) across the experiments in two ways: by distinguishing between experiments with and without effects based on standard statistical criteria, and by using an unsupervised Bayesian method to fit their distributions.

### Estimating Effect Sizes From the Data Sets

#### SDs of between-condition LT differences

**Figure 3 fig3:**
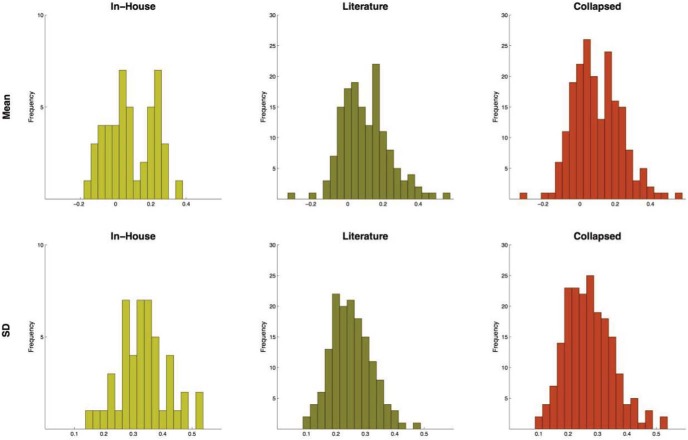
Histograms of the means and *SD*s of log-looking time (LT) differences in the two data sets separately and together. See the online article for the color version of this figure.

The average *SD* of the base-10 log-transformed LT differences across studies in the in-house data set was 0.332. This is higher than the average *SD* of log-transformed LTs (0.298) because the average correlation between the log-transformed LTs in the two conditions was 0.380 (lower than 0.5). The between-condition differences were not directly available in the literature data set, but were estimated from *t*-values and from assumed levels of correlations (see above). With these estimates, we found that the average *SD* of LT differences across conditions in this data set was 0.252, significantly lower than what we found in the in-house data set (*t*_194_ = 6.583, *p* < .001). This was partly due to the fact that in the literature data set auditory studies produced lower *SD*s of LT difference (0.223) than visual ones (0.272; *t*_147_ = 4.486, *p* < .001). Nevertheless, collapsing the two data sets did not create a visibly bimodal distribution (Figure 3, bottom row, right column). In the aggregated data set of 196 experiments, the average *SD* of LT differences was 0.271.[Fig-anchor fig3]

#### Means of between-condition LT differences

The top row of Figure 3 depicts histograms of the size of the average between-condition difference in the two data sets separately and together. The distribution of these differences in the in-house data set is clearly bimodal with a peak around 0.04, representing the experiments that did not produce differential LTs, and another peak around 0.24, representing those that might have revealed an effect. Note that not all experiments that fell around 0.2 produced statistically significant results—some might have failed this test because of higher *SD*s (or lower sample sizes). Among the 18 experiments that, after log-transformation, actually produced a statistically significant difference by conventional *t* tests, the average between-condition difference was 0.216 (64.2% increase or 39.1% decrease of LT in raw value). The data set collected from the literature also produced a bimodal distribution of between-condition differences, with peaks around 0.04 and around 0.16 (Figure 3, top row, middle column). Of the 71 experiments that, according to our estimates, would have resulted in significant effects in the predicted direction had they been analyzed with log-transformed data, the average significant difference was 0.210.

The average LT difference between conditions in the experiments that produced (or would have produced) a statistically significant effect at 0.05 level was not significantly different between the data sets (*t*_88_ = 0.325) or between auditory and visual studies (*t*_88_ = 0.071), and was not significantly correlated with the age group in which the experiment was performed. In the collapsed data set the average between-condition effect in successful experiments was 0.210, while the experiments without an effect produced an average difference value of 0.000. We conclude that a suitable estimate for the mean between-condition difference for a generic successful LT study would be around 0.2. This value corresponds to a 58.5% of increase (or 36.9% of decrease, if the higher LT is considered to be the baseline) of raw LTs between conditions.

Note that this last analysis has two questionable elements that we rectify in the next section. First, it requires a hard categorical decision to be made about whether an experiment did or did not produce an effect, which can only be made using an arbitrary threshold for significance (here, at 0.05). This can also lead to “double dipping” (Kriegeskorte et al., 2009), that is, making the analysis circular when some data (here, LT difference) is used to create categories (presence or absence of effect), and then this categorical judgment is used to select samples to estimate some statistic of the same data (here, the mean of LT differences) in each category. Second, taking a plain average across studies (of one category) ignores the fact that the sample size of the experiments included in the analysis were different (ranging 9 to 59), and so the reliability of these experiments with which mean LT differences (and *SD*s) reflected the underlying populations’ mean (and *SD*) was also different.

### Model-Based Analyses

Another approach to the estimation of the parameters of log-transformed LT difference data is fitting explicit probabilistic models to the available data. Unlike the analyses of the previous section, the procedure by which we determined the parameters of our models did not require a prior hard categorical judgment as to which experiment was deemed to produce an effect. Instead, the analyses inferred this information probabilistically at the same time while estimating the model parameters in a completely unsupervised way, by simply maximizing the likelihood of the model on the given data set (Bishop, 2006). Moreover, we formulated our models at the level of individual participants’ LT differences, which ensured that experiments with larger sample sizes contributed more to the parameter estimates, as appropriate. (For the details of the models and the fitting procedure, see Appendix C.)

We adopted two approaches to model the data. The first approach took the empirical description of the findings in terms of a bimodal distribution of LT differences (see the previous section) at face value, and assumed that the distributions of experiments with and without a real effect differ in means but not in variance, and all successful experiments had the same underlying LT difference. In contrast, the second approach assumed that the underlying LT differences of experiments with real effects can be different and themselves vary around zero, and the within-experiment variance can be different for experiments with and without an underlying effect.

#### Fixed LT difference

This model assumed that LT differences were coming from two normal distributions having the same variance, but different means: one had a positive mean, which was unknown and thus had to be estimated from the data, and the other had a mean of zero (respectively, corresponding to experiments producing or failing to produce an effect). Note that this model assumed that all experiments within either category had the same expected LT difference and the empirical variation in the actual sample means was entirely because of limited sample sizes. After fitting this model, we found that in the in-house data set the mean of the positive distribution (i.e., the distribution of the experiments that produced an effect) was 0.210 and the *SD* of the LT differences was 0.345. These values were very close to our previous estimates (0.216 and 0.332, respectively). The mean of the positive LT difference (0.209) and *SD* (0.264) yielded by the model for the literature data set also matched well our previous estimates (0.210 and 0.252, respectively). The best fit distribution for the data collapsed across data sets had a mean of 0.209 and *SD* of 0.296.

#### LT differences varying around zero

This model assumed that subjects’ LT differences were coming from two types of normal distributions: (a) the experiments without an underlying effect were modeled with a normal distribution with zero mean and unknown variance, and (b) the experiments with an effect were modeled by a normal distribution with an unknown variance and a mean which itself was assumed to vary across studies and to be distributed normally with zero mean and unknown variance. This model thus assumed that different “successful” experiments may have fundamentally different effect sizes. While the previous approach estimated two parameters (the LT difference and the equal variance of the two distributions), this model estimated three parameters: the variance of the LT means around zero in experiments with an effect, and the variances of LT differences within experiments with and without an effect separately. We estimated these parameters only for the collapsed data sets. The analysis yielded the best fit with the data where the across-experiments *SD* of the mean LT difference was 0.171, and the *SD* of the within-experiment LT differences was 0.325 and 0.195 for experiments with and without effects, respectively. This model provided a better fit with the data than the previous one: it produced a Bayesian information criterion (BIC; Bishop, 2006; see footnote 1 for formula) 220.88 as opposed to the BIC of the previous model, which was 177.78, thus corresponding to a factor of 10^43^ difference in their likelihoods.

### Interpretation

Our various estimates of the log-transformed LT differences in studies exhibiting an effect across conditions ranged from 0.209 to 0.216, while the SDs of these differences ranged from 0.223 (in auditory studies) to 0.332 (in the in-house data set). We propose that the suitable estimate for these parameters for a typical LT study would be 0.2 for the expected mean and 0.3 for its *SD*. Such values would yield an effect size of 0.667, which would require at least 12 participants to produce a statistically significant effect with a *t* test at α = 0.05.

While these statistical parameters describe well the experiments included in our data sets, these studies may not be representative of all possible LT studies. Indeed, it is possible that the sets of studies we used in our analyses is biased by selective publication (experiments with small effects and small sample size may not appear in articles) and by the particular topics investigated. Thus, the bimodal distribution of effect sizes may be artifactual, and should be taken with caution when it is exploited to generate expectations for future studies. In this respect, the parameter estimates from the second model-based analysis may provide safer predictions for LT studies that are different from the ones we selected for our analyses. On the basis of this model, we would estimate that experiments with underlying effects produce LT differences that vary around zero with *SD* of 0.2 and had a between-subjects *SD* of 0.3 (matching the previous estimate), while the experiments without an effect display a smaller between-subjects *SD* of 0.2. We use these estimates to develop Bayesian statistics for LT experiments in the next section.

## Bayesian Statistics of LTs

Bayesian statistics offer various advantages over traditional “frequentist” tests, including the possibility to infer the absence of effects, no need to prefix sample size, and so forth (Dienes, 2011, 2014; Gallistel, 2009; Glover & Dixon, 2004; Kruschke, 2011; Morey & Rouder, 2011). However, the most popular Bayesian statistical methods, which rely on determining Bayes factors calculated as the ratio between the likelihood of two competing hypotheses that could explain the data, require the researcher to provide quantitative estimates of the size of the expected effect under each hypothesis.

In most LT studies, the two competing hypotheses can be conceived as the one (*H*_1_) according to which the experimental manipulation (i.e., the difference between conditions) exerts an effect on the dependent measure (i.e., on LT), and the one (*H*_0_) according to which no such effect occurs. Note that while the size of the expected effect under *H*_0_ is explicit, that is, it predicts zero difference between LTs in the two conditions, *H*_1_ remains unquantified in traditional statistical approaches. Calculating Bayes factors requires the specification of how much the LTs should increase (or decrease) in one condition compared to the other. Using raw LT data, this is rarely a viable method because the size of the increase also depends on the baseline LT measure.

However, because log-transformed differences essentially measure multiplicative effects, they are not scaled with the baseline value. Thus, if a researcher performs a LT experiment and predicts an average sized effect for her experimental manipulation, she could hypothesize expected effect size estimation from other LT studies. The best strategy to obtain such an effect size would be to base the estimate on previous data with similar methods. For example, one could estimate this effect size by relying on data from previous studies applying similar stimuli, similar age groups, and similar overall methods (e.g., familiarization or habituation). However, our analysis suggests that the log-transformed LT differences between conditions do not vary tremendously across studies, which will enable researchers to form effect size estimates even in the absence of prior studies in their specific domain.

We developed two ways of performing Bayesian analysis on LT data. The first one assumes that the average effect size we identified in our data sets provides a suitable *H*_1_ for any potential future LT study. The second type of analysis relies on the results of the model-based analysis that assumed varying effect sizes. We investigated what conclusions one could draw from the experiments on our samples with each of these Bayesian analyses by calculating the corresponding Bayes factors. Following conventions (Dienes, 2011; Jeffreys, 1961), we judged the evidence to be substantially or strongly in favor of the theory that the manipulation had an effect on LTs if the Bayes factor was larger than 3 or 10, respectively. Instead of straight Bayes factors, we calculated and report here their base-10 logarithm, which above 1.0 would indicate strong, and above 0.5 would indicate substantial effects. Conversely, when the logarithm of the Bayes factor (lBF henceforth) falls below −1 (or −0.5), we consider the contrastive hypothesis (i.e., that there is no effect) to be favored strongly (or substantially) by the evidence.

### Bayesian Analysis Assuming Fixed Effect Size

Using the values that we derived from the two data sets we analyzed, one could predict that the between-condition differences of log-transformed LTs in an experiment with averaged-sized (i.e., neither too weak, nor not too strong) expected effect will come from a normal distribution with the mean of 0.2 and *SD* of 0.3 (these values assume base-10 log-transformation). We calculated lBFs for each experiment contrasting two hypotheses predicting that the experimental manipulation would result in a log-LT difference that comes (*H*_1_) from a normal distribution with the mean of 0.2 and *SD* of 0.3, or (*H*_0_) from a normal distribution with zero mean and the same *SD*. We calculated the base-10 lBF the following way (for derivation, see Appendix D):

$$\text{lBF}={\text{log}}_{\text{1}0}\left(e\right)\times n\times \mu \times (m-\mu /\text{2})/\sigma^{\text{2}},$$ 1

where *e* is the base of the natural logarithm, *n* is the sample size, *m* is the mean of the differences of log-transformed LTs in the sample, and μ and σ are the mean and *SD* of the hypothesized distribution of the experimental effect. With μ = 0.2 and σ = 0.3, this calculation is further simplified to lBF = 0.965 × *n* × (*m* − 0.1) ≈ *n* × (*m* − 0.1). This Bayes factor acts like a one-sided test: it will show high positive values only when the effect occurs in the predicted direction. In case *m* is negative and the above lBF is strongly negative (e.g., lBF < −1.0), one can also test whether the opposite LT effect is obtained by calculating lBF as −0.965 × *n* × (*m* + 0.1).

#### In-house data set

In 17 of the 18 experiments judged by conventional statistical analyses to have resulted in significant effects in the predicted direction this verdict was confirmed by the Bayesian analysis. In three of these experiments, the lBF values were between 0.5 and 1.0 (substantial evidence), in the other 14 experiments they went up to 3.98 (corresponding to a Bayes factor close to 10,000), indicating strong evidence for the predicted effect. The experiment in which the Bayesian statistics did not confirm the significance of the effect displayed a very small looking time difference (below 0.1 in base-10 log units). However, the results of two experiments that failed to reach the level of statistical significance with conventional statistics indicated very strong effects (lBFs above 2.0) in Bayesian analyses. An additional 22 control experiments, which showed no effect with conventional statistics, plus the single experiment that resulted in LT difference opposite to the predicted one, provided evidence for the null effect (lBFs below −0.5).

#### Literature data set

In the 71 experiments for which conventional statistics on the estimated log-transformed data judged the predicted effect statistically significant, the lBFs ranged from −1.607 to 12.272. Eleven of these values were below 0.5, indicating only weak evidence for the effect of experimental manipulation by Bayesian statistics, and 46 of them were above 1.0, indicating strong evidence. Thus, in 60 of 71 cases the Bayesian statistics confirmed the result of conventional statistics. In two further experiments, where a difference was predicted by researchers but their prediction was not confirmed by conventional statistics, Bayesian analysis suggested substantial evidence supporting this prediction. In addition, Bayesian analyses would have allowed researchers to infer the absence of effect in many control experiments in the literature data set (see the Supplementary Material).

### Bayesian Analysis Assuming Variable Effect Size

The second model-based analysis suggested that the LT differences in experiments with an underlying effect may vary around zero with an *SD* of 0.2, and the within-experiment (between-subjects) *SD* could be estimated as 0.3 for experiments with an effect and as 0.2 for experiments without an effect. The lBFs based on this model can be calculated using the following approximate formula (for derivation, see Appendix D):

$$\begin{array}{c} \text{lBF}≃\left[{\text{log}}_{\text{1}0}\left(e\right)\times \left(\text{1}/\beta -\text{1}/\alpha \right)\times n\times m^{\text{2}}-{\text{log}}_{\text{1}0}\left(\alpha /\beta \right)\right]/\text{2},\\ \text{where}\;\alpha =\sigma_{1}^{2}+n\times \omega_{1}^{2}\;\text{and}\;\beta =\sigma_{0}^{2}, \end{array}$$ 2

where *e* is the base of the natural logarithm, *n* is the sample size, *m* is the mean of the differences of log-transformed LTs in the sample, ω_1_ is the *SD* of the mean LT differences, and σ_0_ and σ_1_ are the *SD*s of the hypothesized distribution without and with an effect, respectively. Substituting ω_1_ = 0.2, σ_0_ = 0.2, and σ_1_ = 0.3, the calculation becomes

$$\text{lBF}≃\left[\text{5}.\text{429}-\text{21}.\text{715}/\left(\text{9}+\text{4}n\right)\right]\times n\times m^{\text{2}}-{\text{log}}_{\text{1}0}\left[\surd \left(\text{9}+\text{4}n\right)\right]+0.\text{3}0\text{1}.$$ 3

In this case, the Bayesian analysis acts as a two-sided test: high positive lBF will be obtained regardless of whether the LT differences are strongly above or below zero, while negative lBF values support the conclusion of no effect in either direction.

#### In-house data set

Just like in the previous Bayesian analyses, the lBFs confirmed the results of the conventional statistics in all but one experiment. In fact, these analyses yielded considerably higher Bayes factors than the earlier ones. In addition, a high positive lBF (1.149) was found for the experiment that resulted in the opposite looking time difference to what had been predicted. The two experiments that produced high Bayes factors in the earlier analyses despite failing to reach the level of statistical significance with conventional statistics again produced high lBFs. However, only nine of the remaining experiments carried some evidence for *H*_0_, and none of these was strong (−1.0 < lBFs < −0.5).

#### Literature data set

The results of these Bayesian analyses were even more consistent with those of the conventional analyses than the previous one. Only five (of 71) experiments that conventional statistics on the estimated log-transformed data judged the predicted effect statistically significant did not produce substantial evidence for *H*_1_ on the basis of lBFs. In addition, *H*_1_ was supported in four experiments that did not produce a significant effect in *t* tests, and in four further experiments an effect to the opposite direction was confirmed. This type of Bayesian analysis would have confirmed the absence of the effect (i.e., confirmed *H*_0_) in 37 experiments.

### Comparing Bayesian Analyses

The two versions of Bayesian analyses differed in how *H*_1_ was specified. Nevertheless, in most experiments from both sets of studies the conclusions that the two types of Bayesian statistics supported agreed with each other (for a study-by-study comparison, see the Supplementary Material). When *H*_1_ assumed a fixed effect size, the analyses produced less positive (and more negative) lBFs than when *H*_1_ hypothesized variable effect sizes. Thus, the former analysis were more likely to support *H*_0_ and less likely to support *H*_1_ than the latter one.

## Recommendations

On the basis of our analyses of the two data sets, we offer the following recommendations for researchers who intend to perform experiments measuring LTs in infants.

### Logarithmic Transformation

Looking time data should be subjected to logarithmic transformation before statistical analysis, whether or not the data collected are found to violate the assumption of normality by a statistical test. Note that this requirement assumes that the LT data are on a ratio scale (rather than on an interval scale) with a suitably chosen zero point (e.g., the moment when the information to be processed becomes available).

### Sample Size

For experiments that are intended to be analyzed by conventional statistics, the sample size should be fixed in advance. Assuming that the parameters we derived from the two sets of studies are representative, the effect of a within-subject independent variable on log-transformed LTs will have sufficient power to demonstrate an effect with two-tailed *t* tests (α = 0.05) with 0.75 probability by 16 participants, and with 0.95 probability by 32 participants. If the expected effect is smaller than usual, that is, if the expected increase of LT from the lower to the higher value is between 40 and 50%, we recommend a minimum sample size of 26 to achieve a power of 0.75. An experimental manipulation that is expected to increase raw LTs by less than 35% with normal between-subjects variance is unlikely to be successful if the sample size is smaller then 40.

### Bayesian Analyses

In the absence of previous experiments with similar design or available theoretical considerations, a new study can hypothesize (*H*_1_) that the base-10 log-transformed LTs increase or decrease by an unknown amount that comes from a normal distribution around 0 with an *SD* of 0.2, while the within-study *SD* of LT differences would be 0.3. A suitable *H*_0_ against this prediction is that the log-LT differences will come from a normal distribution with a mean of zero and an *SD* of 0.2. These values are robust—they work with a wide range of experiments and actual effect sizes—and the corresponding approximate lBF values are easy to calculate for any sample by Equations 2-3 above.

Alternatively, if previous studies allow the development of a *H*_1_ for a specific effect size, one can adopt the method we followed above for fixed effect sizes. Equation 1 can be used for calculating lBFs with any hypothesized LT difference as long as the predicted *SD* of the difference is the same for *H*_1_ and *H*_0_.

Experiments analyzed by Bayes factors do not have to fix the sample size in advance. Instead, the researcher could check periodically whether the collected data provides sufficient evidence for either that the manipulation works (e.g., by obtaining an lBF value larger than 1.0) or that it does not work (lBF below −1.0). For example, if counterbalancing some variables demands a sample in multiples of 8 participants, one could calculate the Bayes factor at the 8th, 16th, 24th, and so forth, participant, and stop the experiment when the data provide sufficiently strong evidence for either the presence or the absence of the effect of the manipulation, or otherwise terminate it without a verdict when the sample size reaches a high limit (e.g., 40 participants). While the method of expanding the sample size until a desirable effect is found (“preferential stopping”) would invalidate conventional “frequentists” statistical analyses, it is permissible with Bayesian analysis (Dienes, 2011). Although, in certain situations, preferential stopping could also produce high Bayes factors with unreasonably high probability, this mostly affects Bayes factors favoring the null hypothesis (false negatives), so that the probability of obtaining a spuriously high Bayes factor supporting the alternative hypothesis (false positive, the concern of standard frequentist statistical analyses) still remains bounded (Sanborn & Hills, 2014).

## Conclusions

Our analyses were restricted to the between-subjects statistical properties of LTs, and do not allow us to evaluate or judge the methodological soundness or scientific value of the studies in either data set. Nevertheless, our recommendations can benefit future studies by allowing researchers to justify their choice of analysis. Currently, log-transformation is hardly applied to LT data in the literature (e.g., none of the studies in the literature data set used it), and when it is applied, the researchers feel compelled to justify this step by referring to the skewness of the data or to a test of normality. Our analyses suggest that *not* applying log-transformation is the methodological choice that should require justification.

When a new LT study is designed, the researchers need to decide how they will statistically analyze the collected data. Both conventional and Bayesian analyses require predetermining some parameters (the sample size and the effect size, respectively) before conducting the experiment. Our recommendations offer researchers justifications for choosing these parameters for LT studies even when theoretical or empirical considerations do not allow prior specification of them in the given field of research.

Considering how simple technique it is, measuring looking times has become a very popular and flexible tool in infant research. Improving the analyses of collected LT data will make it even more valuable.

## Supplementary Material

10.1037/dev0000083.supp

## Figures and Tables

**Table 1 tbl1:** List of Studies in the “In-House” Data Set

Study	Number of experiments	Age range (months)	Participants per experiment
Hernik, M. (unpublished). Self-steering and goal-attribution in 12-month-olds.	1	12	24
Hernik, M., & Csibra, G. (2015). Infants learn enduring functions of novel tools from action demonstrations. *Journal of Experimental Child Psychology, 130,* 176–192. (Experiment 1–3, and a pilot study)	4	12–14	16
Hernik, M., Fearon, R. M. P & Southgate, V. (in preparation). Goal-attribution in 6-months-old infants critically depends on action efficiency.	2	6	16
Hernik, M. & Haman, M. (in preparation). Fourteen-month-olds transfer sequences of features derived from internally-driven object transformation.	3	14	16
Hernik, M., & Southgate, V. (2012). Nine-months-old infants do not need to know what the agent prefers in order to reason about its goals: on the role of preference and persistence in infants’ goal-attribution. *Developmental Science,15,* 714–722.	3	9	16
Mascaro, O. & Csibra, G. (2012). Representation of stable dominance relations by human infants. *Proceedings of the National Academy of Sciences of the USA, 109,* 6862–6867.	8	9–15	16
Mascaro, O. & Csibra, G. (2014). Human infants’ learning of social structures: The case of dominance hierarchy. *Psychological Science, 25,* 250–255.	4	15	24
Mascaro, O. & Csibra, G. (in preparation). Fourteen-month-old infants compute the efficiency of joint actions.	2	14	16
Tatone, D., & Csibra, G. (in preparation 1). Infants’ encoding of reciprocity-tracking information is specific for benefit exchanges based on giving.	4	12	16
Tatone, D., & Csibra, G. (in preparation 2). Beyond the triad. Giving—but not taking—actions prime equality expectations in dyadic social interactions for 15-month-old human infants.	3	12–15	16
Tatone, D., & Csibra, G. (unpublished). No evidence of equality expectations for redistributive interactions based on taking actions in 12- and 15-month-olds.	4	12–15	16
Tatone, D., Geraci, A., & Csibra, G. (2015). Giving and taking. Representational building blocks of resource-transfer events in human infants. *Cognition, 137,* 47–62. (Experiments 1–7)	7	12	16
Tatone, D., Hernik, M., & Csibra, G. (in preparation). The side effect that wasn’t. Other-benefiting outcomes crucially influence infants’ goal attribution.	2	15	16

**Table 2 tbl2:** List of Studies in the “Literature” Data Set

Study	Number of experiments	Age range (months)	Participants per experiment
Bahrick, L. E., Lickliter, R., & Castellanos, I. (2013). The development of face perception in infancy: intersensory interference and unimodal visual facilitation. *Developmental Psychology, 49,* 1919–1930.	5	2–3	16
Beier, J. S., & Spelke, E. S. (2012). Infants’ developing understanding of social gaze. *Child Development, 83,* 486–496.	6	9–10	16–20
Bremner, J. G., Slater, A. M., Johnson, S. P., Mason, U. C., & Spring, J. (2012). The effects of auditory information on 4-month-old infants’ perception of trajectory continuity. *Child Development, 83,* 954–964.	6	4	12
Casasola, M., & Park, Y. (2013). Developmental changes in infant spatial categorization: When more is best and when less is enough. *Child Development, 84,* 1004–1019.	1	10	9
Ceulemans, A., Loeys, T., Warreyn, P., Hoppenbrouwers, K., Rousseau, S., & Desoete, A. (2012). Small number discrimination in early human development: the case of one versus three. *Education Research International*. http://dx.doi.org/10.1155/2012/964052	1	8	16
Cheung, H., Xiao, W., & Lai, C. M. (2012). Twelve-month-olds’ understanding of intention transfer through communication. *PLoS ONE, 7,* e46178.	4	12	18
Curtin, S., Campbell, J., & Hufnagle, D. (2012). Mapping novel labels to actions: How the rhythm of words guides infants’ learning. *Journal of Experimental Child Psychology, 112,* 127–140.	3	16	14–20
Daum, M. M., Attig, M., Gunawan, R., Prinz, W., & Gredebäck, G. (2012). Actions seen through babies’ eyes: a dissociation between looking time and predictive gaze. *Frontiers in Psychology, 3,* 370.	1	9	24
Fennell, C. T. (2012). Object familiarity enhances infants’ use of phonetic detail in novel words. *Infancy, 17,* 339–353.	2	14	23–24
Flom, R., & Pick, A. D. (2012). Dynamics of infant habituation: Infants’ discrimination of musical excerpts. *Infant Behavior and Development, 35,* 697–704.	6	5–7	24
Graf Estes, K. G. (2012). Infants generalize representations of statistically segmented words. *Frontiers in Psychology, 3,* 447.	5	11–17	22–28
Graf Estes, K., & Hurley, K. (2013). Infant-directed prosody helps infants map sounds to meanings. *Infancy, 18,* 797–824.	3	18	26–28
Henderson, A. M., & Woodward, A. L. (2012). Nine-month-old infants generalize object labels, but not object preferences across individuals. *Developmental Science, 15,* 641–652.	4	9	20
Hohenberger, A., Elsabbagh, M., Serres, J., de Schoenen, S., Karmiloff-Smith, A., & Aschersleben, G. (2012). Understanding goal-directed human actions and physical causality: The role of mother–infant interaction. *Infant Behavior and Development, 35,* 898–911.	4	6–10	21–59
Ma, L., & Xu, F. (2013). Preverbal infants infer intentional agents from the perception of regularity. *Developmental Psychology, 49,* 1330.	6	9	16
Macchi Cassia, V., Picozzi, M., Girelli, L., & de Hevia, M. D. (2012). Increasing magnitude counts more: Asymmetrical processing of ordinality in 4-month-old infants. *Cognition, 124,* 183–193.	6	4	12
MacKenzie, H., Curtin, S., & Graham, S. A. (2012). Class matters: 12-month—olds’ word—object associations privilege content over function words. *Developmental Science, 15,* 753–761.	3	12	16
Marcus, G. F., Fernandes, K. J., & Johnson, S. P. (2012). The role of association in early word-learning. *Frontiers in Psychology, 3,* 283.	5	7–14	18–20
Marquis, A., & Shi, R. (2012). Initial morphological learning in preverbal infants. *Cognition, 122,* 61–66.	3	11	16
Martin, A., Onishi, K. H., & Vouloumanos, A. (2012). Understanding the abstract role of speech in communication at 12 months. *Cognition, 123,* 50–60.	5	12	16–28
Moher, M., Tuerk, A. S., & Feigenson, L. (2012). Seven-month-old infants chunk items in memory. *Journal of Experimental Child Psychology, 112,* 361–377.	7	7	20
Möhring, W., Libertus, M. E., & Bertin, E. (2012). Speed discrimination in 6-and 10-month-old infants follows Weber’s law. *Journal of Experimental Child Psychology, 111,* 405–418.	3	6–10	24
Muentener, P., Bonawitz, E., Horowitz, A., & Schulz, L. (2012). Mind the gap: Investigating toddlers’ sensitivity to contact relations in predictive events. *PLoS ONE, 7,* e34061.	8	24	22
Ozturk, O., Krehm, M., & Vouloumanos, A. (2013). Sound symbolism in infancy: evidence for sound—shape cross-modal correspondences in 4-month-olds. *Journal of Experimental Child Psychology, 114,* 173–186.	3	4	12
Pons, F., Albareda-Castellot, B., & Sebastián-Gallés, N. (2012). The interplay between input and initial biases: Asymmetries in vowel perception during the first year of life. *Child Development, 83,* 965–976.	12	4–12	12
Schlottmann, A., Ray, E. D., & Surian, L. (2012). Emerging perception of causality in action-and-reaction sequences from 4 to 6 months of age: Is it domain-specific?. *Journal of Experimental Child Psychology, 112,* 208–230.	7	5–6	16–56
Sirois, S., & Jackson, I. R. (2012). Pupil dilation and object permanence in infants. *Infancy, 17,* 61–78.	2	10	19
Sloane, S., Baillargeon, R., & Premack, D. (2012). Do infants have a sense of fairness? *Psychological Science, 23,* 196–204.	6	19–21	16–18
Soska, K. C., & Johnson, S. P. (2013). Development of three-dimensional completion of complex objects. *Infancy, 18,* 325–344.	6	4–9.5	16
Spangler, S. M., Schwarzer, G., Freitag, C., Vierhaus, M., Teubert, M., Fassbender, I., . . . & Keller, H. (2013). The other-race effect in a longitudinal sample of 3-, 6-and 9-month-old infants: Evidence of a training effect. *Infancy,18,* 516–533.	6	3–9	53–54
Ting, J. Y., Bergeson, T. R., & Miyamoto, R. T. (2012). Effects of simultaneous speech and sign on infants’ attention to spoken language. *The Laryngoscope, 122,* 2808–2812.	2	8	10
Vaillant-Molina, M., & Bahrick, L. E. (2012). The role of intersensory redundancy in the emergence of social referencing in 5½-month-old infants. *Developmental Psychology, 48,* 1–10.	2	6	16
Weikum, W. M., Oberlander, T. F., Hensch, T. K., & Werker, J. F. (2012). Prenatal exposure to antidepressants and depressed maternal mood alter trajectory of infant speech perception. *Proceedings of the National Academy of Sciences of the USA, 109,* 17221–17227.	6	6–10	16–30

## Footnotes

1 We also compared directly whether a Normal (N), log-normal (LN), shifted-LN (sh-LN), ex-Gaussian (ex-G, the sum of an exponential and a normal random variable), or γ distribution fitted better the data in this data set. Using the Bayesian Information Criterion (BIC = log_10_(likelihood with optimized parameters) − 1/2 × number of parameters × log_10_(#data points); Bishop, 2006) as a measure of goodness-of-fit, we found that the data were overwhelmingly in favor of the LN distribution, which was 10^126^-times more likely than the N, 10^37^-times more likely than the sh-LN, 10^21^-times more likely than the ex-G, and 10^5^-times more likely than the γ distribution.

2 The average correlation coefficient between LTs to the two conditions (*r*_R_) calculated on the untransformed data in the in-house data set was 0.414. This was reduced to 0.380 after log-transformation (*r*_L_). We used the regression equation between these two sets of correlation coefficients (*r*_L_ = 0.8177 × *r*_R_ + 0.0414) for estimating the correlation coefficients for the log-transformed LTs in the literature data set.

## Appendix A Estimating the Parameters of Log-Transformed Data

Estimating the mean (*m*_t_) and *SD* (*s*_t_) of base-10 log-transformed data from the mean and *SD* of raw data assumed to come from a log-normal distribution.

$$m_{\text{t}}={\text{log}}_{\text{1}0}\left(m^{\text{2}}/{\left[m^{\text{2}}+s^{\text{2}}\right]}^{½}\right)$$

$$s_{\text{t}}={\left({\text{log}}_{\text{1}0}\left(e\right)\times {\text{log}}_{\text{1}0}\left[\text{1}+s^{\text{2}}/m^{\text{2}}\right]\right)}^{½}$$

where *m* and *s* are the mean and *SD* of the raw data and *e* is the base of the natural logarithm.

## Appendix B Using *t* Values to Calculate ***SD***s and Correlations

Calculating the correlation coefficient of LTs and the *SD* of LT differences from the available data.

Available:

*n*: Sample size
*m*_1_ and *m*_2_: Mean LTs in the two conditions
*s*_1_ and *s*_2_: The *SD*s of LTs in the two conditions
*t*: The value of the *t* statistics

Calculated:

*r*: Pearson correlation between conditions
*s*_d_: The SD of the LT difference between conditions

Since

$$t=\surd n\times \left(m_{\text{1}}-m_{\text{2}}\right)/s_{\text{d}}$$

$$s_{d}^{2}=n\;\times {\left(m_{\text{1}}-m_{\text{2}}\right)}^{\text{2}}/t^{\text{2}}$$ B1

*s*_d_^2^ can also be expressed from the two known *SD*s:

$$s_{\text{d}}^{2}=s_{1}^{2}+s_{2}^{2}-\text{2}\times r\times s_{\text{1}}\times s_{\text{2}}$$ B2

From (B2):

$$r=\left(s_{1}^{2}+s_{2}^{2}-s_{d}^{2}\right)/\left(\text{2}\times s_{\text{1}}\times s_{\text{2}}\right)$$

Substituting *s*_d_^2^ from (B1):

$$r=\left(s_{1}^{2}+s_{2}^{2}-\left[n\times {\left(m_{\text{1}}-m_{\text{2}}\right)}^{\text{2}}/t^{\text{2}}\right]\right)/\left(\text{2}\times s_{\text{1}}\times s_{\text{2}}\right)$$

## Appendix C Model-Based Estimation of the Statistical Parameters of Log-LT Difference Distributions

### Model-based estimation assuming fixed effect size

We modeled individual participants’ log-LT differences as coming from one of three normal distributions having the same *SD* (σ), one with zero mean (absence of effect), the other with a positive mean (μ, presence of effect), and the third with a negative mean of the same magnitude (−μ, presence of effect, but in the opposite direction to what was expected).

As we did not have access to individual participants’ data in some cases (in particular, in the literature data set), we derived model predictions for the summary statistics (sample mean and *SD*) of the data to which we always had access. For this, all participants of the same experiment were assumed to come from the same normal distribution, hence the joint distribution of the sample mean and variance of their log-LT differences were statistically independent given the parameters of the underlying normal distribution, with the sample mean being distributed normally with mean = 0, μ, or −μ, and *SD* = σ/√*n*, where *n* is the sample size (number of participants), and the sample variance (*SD* squared) being γ distributed with shape parameter (*n* − 1)/2 and scale parameter 2σ^2^/(*n* − 1). Furthermore, we treated the indicator variable, indicating whether an experiment did or did not produce an effect, and if so in which direction (i.e. whether the participants’ data came from the zero-, the positive-, or the negative-mean normal distribution), as a hidden variable and properly integrated it out, with a prior probability of it belonging to each of these categories, *p*_0_, *p*_1_, and *p*_2_ = 1 − *p*_0_ − *p*_1_, respectively.

In summary, the unknown parameters that needed to be estimated from data were σ, μ, *p*_0_, and *p*_1_. We estimated these parameters by jointly maximizing their (log) likelihood on the data (set of sample sizes, means and *SD*s in either the in-house, or the literature data set, or both, see main text), adapting the standard expectation-maximization algorithm (Dempster, Laird, & Rubin, 1977) to this case. To avoid overfitting, we placed very weak priors on the *p* parameters (Dirichlet with parameter 1.02) and σ^2^ (inverse γ distribution, with shape parameter 1, and scale parameter 1; Bishop, 2006).

### Model-based estimation assuming variable effect size

We proceeded as in the previous analysis with the following changes. We modeled individual participants’ log-LT differences as coming from one of *two* (rather than three) normal distributions. One distribution (absence of effect) had zero mean and finite SD (σ_0_), and thus the sample statistics of the first type of experiment were distributed as before. The other distribution had a *different* (rather than the same) *SD* (σ_1_), and a mean that was unknown and assumed to *vary* (rather than being identical) across experiments such that it itself was normally distributed with zero mean and some finite *SD* (ω_1_). Note that this implied that sample means for the second type of experiment were normally distributed with zero mean and a variance of σ_1_^2^/*n*+ω_1_^2^ (while the sample *SD* were still distributed as before). As the indicator variable for the type of an experiment could only take on two values, only one parameter was associated with it, the prior probability of an experiment producing an effect, *p*. In summary, the unknown parameters that needed to be estimated from data were σ_0_, σ_1_, ω_1_^2^, and *p*. These were estimated using the same maximum likelihood procedures as before.

## Appendix D Calculation of Bayes Factors

### Bayes factor assuming fixed effect size

The Bayes factor measures the ratio of the likelihood of two hypotheses (Bishop, 2006). In our case, the two relevant hypotheses were whether the participants’ log-LT differences in a new experiment come from the zero-mean (absence of effect) or positive-mean (presence of effect) normal distribution. (If they are more likely to come from the zero mean then one can analogously further measure the Bayes factor of the negative- vs. zero-mean hypotheses.) This can be formalized as

$$\begin{array}{l} \text{BF}=\;(\text{Normal}\left[m;\;\mu ,\sigma /\surd n\right]\times \text{Gamma}\left[s^{\text{2}};\;\left(n-\text{1}\right)/\text{2},\text{2}\sigma^{\text{2}}/\left(n-\text{1}\right)\right])/\\ \;(\text{Normal}[m;\;0,\sigma /\surd n]\times \text{Gamma}[s^{\text{2}};\;\left(n-\text{1}\right)/\text{2},\text{2}\sigma^{\text{2}}/\left(n-\text{1}\right)]) \end{array}$$

where *m* and *s* are the sample mean and *SD* of the new data, and μ and σ are set by the hypothesized effect size, determined, for example, by model fitting to previous data sets (see Appendix C). As one can see, the terms containing the sample *SD* (*s*) cancel between the numerator and the denominator, and so one arrives at a much simpler formula:

BF=Normal[m;μ,σ/√n]/Normal[m;0,σ/√n]

of which the base-10 logarithm can be expressed as

$$\text{lBF}={\text{log}}_{\text{1}0}\left(\text{e}\right)\times n\times \mu \times (m-\mu /\text{2})/\sigma^{\text{2}}$$

where *e* is the base of the natural logarithm as above.

### Bayes factor assuming variable effect size

The Bayes factor is computed as above with the following changes. Its formula becomes:

$$\begin{array}{l} \text{BF}=\;\;(\text{Normal}\left[m;\;0,\surd \left(\sigma_{1}^{2}/n+\omega_{1}^{2}\right)\right]\times \text{Gamma}\left[s^{\text{2}};\left(n-\text{1}\right)/\text{2},\text{2}\sigma_{1}^{2}/\left(n-\text{1}\right)\right])/\\ \;(\text{Normal}[m;\;0,\sigma_{0}/\surd n]\times \text{Gamma}[s^{\text{2}};\left(n-\text{1}\right)/\text{2},\text{2}\sigma_{0}^{2}/\left(n-\text{1}\right)]) \end{array}$$

Note that unlike in the previous analysis, the terms containing the sample *SD* (*s*) do not cancel between the numerator and denominator, as they now include different population *SD*s (σ_0_ vs. σ_1_). However, to prevent sample *SD*s driving statistical judgement and to keep the final formula simple and more consistent with that obtained in the previous analysis we still neglect these terms in the following. After a couple of lines of algebra, this results in the following approximate formula for the lBF:

$$\text{lBF}≃\left[{\text{log}}_{\text{1}0}\left(e\right)\times \left(\text{1}/\sigma_{0}^{2}-\text{1}/\left(\sigma_{1}^{2}+n\times \omega_{1}^{2}\right)\right)\times n\times m^{\text{2}}-{\text{log}}_{\text{1}0}\left(\left(\sigma_{1}^{2}+n\times \omega_{1}^{2}\right)/\sigma_{0}^{2}\right)\right]/\text{2}$$

We have made the Matlab code computing these quantities available at https://bitbucket.org/matelengyel/lookingtimes.
